# Poly[diaqua-μ_2_-oxalato-di-μ_4_-succinato-diyttrium(III)]

**DOI:** 10.1107/S1600536809032085

**Published:** 2009-08-19

**Authors:** Zhi-Feng Li, Chun-Xiang Wang, Ping Wang

**Affiliations:** a School of Materials and Chemical Engineering, Jiangxi University of Science and Technology, Ganzhou 341000, People’s Republic of China

## Abstract

In the title compound, [Y_2_(C_4_H_4_O_4_)_2_(C_2_O_4_)(H_2_O)_2_]_*n*_, the flexible succinate anion assumes a *gauche* conformation and bridges the eight-coordinated Y atoms, generating two-dimensional layers parallel to (010). The coordination polymer layers are linked into a three-dimensional framework by the rigid oxalate ligands. The oxalate ions are located on a center of inversion. Inter­molecular O—H⋯O hydrogen bonds help to stabilize the crystal structure.

## Related literature

The title compound is isostructural with [Nd_2_(C_4_H_4_O_4_)_2_(C_2_O_4_)(H_2_O)], see: Wang *et al.* (2007 ▶). For bond lengths and angles in succinate anions, see: Seguatni *et al.* (2004 ▶).
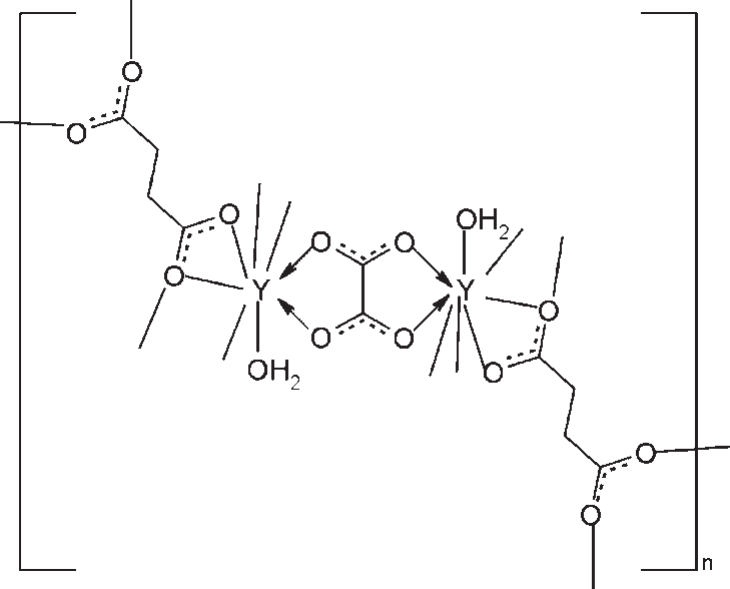


## Experimental

### 

#### Crystal data

[Y_2_(C_4_H_4_O_4_)_2_(C_2_O_4_)(H_2_O)_2_]
*M* *_r_* = 534.02
Triclinic, 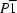

*a* = 6.610 (2) Å
*b* = 7.689 (3) Å
*c* = 8.018 (3) Å
α = 101.589 (5)°
β = 101.843 (4)°
γ = 101.492 (5)°
*V* = 378.2 (2) Å^3^
*Z* = 1
Mo *K*α radiation
μ = 7.71 mm^−1^
*T* = 295 K
0.21 × 0.18 × 0.09 mm

#### Data collection

Bruker APEXII CCD area-detector diffractometer
Absorption correction: multi-scan (*SADABS*; Sheldrick, 2003 ▶) *T*_min_ = 0.215, *T*_max_ = 0.505
2108 measured reflections
1482 independent reflections
1376 reflections with *I* > 2σ(*I*)
*R*_int_ = 0.016

#### Refinement

*R*[*F* ^2^ > 2σ(*F* ^2^)] = 0.022
*wR*(*F* ^2^) = 0.059
*S* = 1.08
1482 reflections
119 parameters
H-atom parameters constrained
Δρ_max_ = 0.60 e Å^−3^
Δρ_min_ = −0.54 e Å^−3^

### 

Data collection: *APEX2* (Bruker, 2004 ▶); cell refinement: *SAINT* (Bruker, 2004 ▶); data reduction: *SAINT*; program(s) used to solve structure: *SHELXS97* (Sheldrick, 2008 ▶); program(s) used to refine structure: *SHELXL97* (Sheldrick, 2008 ▶); molecular graphics: *SHELXTL* (Sheldrick, 2008 ▶); software used to prepare material for publication: *SHELXTL*.

## Supplementary Material

Crystal structure: contains datablocks I, global. DOI: 10.1107/S1600536809032085/jj2005sup1.cif

Structure factors: contains datablocks I. DOI: 10.1107/S1600536809032085/jj2005Isup2.hkl

Additional supplementary materials:  crystallographic information; 3D view; checkCIF report

## Figures and Tables

**Table 1:** Selected bond lengths (Å)

Y—O1	2.4755 (18)
Y—O1^i^	2.3319 (19)
Y—O2	2.4658 (19)
Y—O3^ii^	2.303 (2)
Y—O4^iii^	2.218 (2)
Y—O5	2.3876 (19)
Y—O6^iv^	2.3583 (19)
Y—O7	2.391 (2)

Symmetry codes: (i) 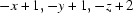; (ii) 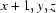; (iii) 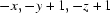; (iv) 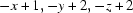.

**Table 2:** Hydrogen-bond geometry (Å, °)

*D*—H⋯*A*	*D*—H	H⋯*A*	*D*⋯*A*	*D*—H⋯*A*
O7—H7*A*⋯O2^v^	0.85	2.02	2.867 (5)	175
O7—H7*B*⋯O5^ii^	0.85	1.96	2.812 (4)	175

Symmetry codes: (ii) 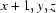; (v) 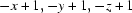.

**Fig. 1.**
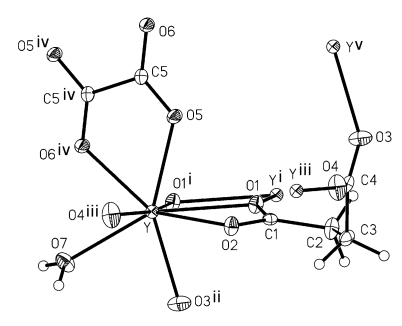
The structure of the title compound, showing the atom-numbering scheme. Displacement ellipsoids are drawn at the 30% probability level and H atoms are shown as small spheres of arbitrary radii. Symmetry code: (i) 1 - x, 1 - y, 1 - z; (ii) x + 1, y, z; (iii) -x, 1 - y, 1 - z; (iv) 1 - x, 2 - y, 2 - z; (v) x - 1, y, z.

**Fig. 2.**
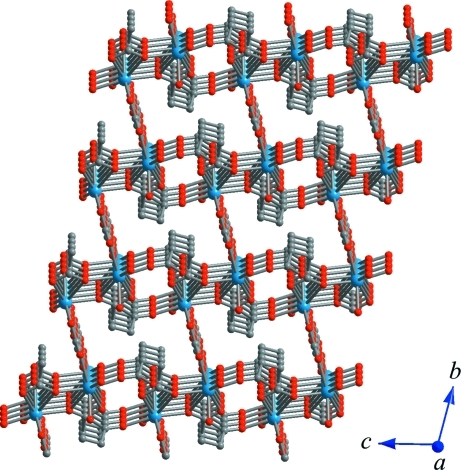
The three-dimensional framework of the title compound.

## supplementary crystallographic information

### Comment

The title compound (I), is isostructural with [Nd_2_(C_4_H_4_O_4_)_2_(C_2_O_4_)(H_2_O)] [Wang et al., 2007]. As shown in Fig.1, the asymmetric unit consists of one Y^3+^ cation, one succinate anion, a half of oxalate anion and one aqua ligand. The Y atoms are each coordinated by eight oxygen atoms of four succinate anions, one oxalate anion and one aqua ligand to complete a distorted square antiprismatic geometry. The Y-O distances range from 2.218 (2) to 2.4755 (18) Å.

In (I), the succinate anions assume a gauche conformation, in which both carboxylate groups exhibit different coordination modes: a common bidentate bridging mode and a tridentate chelating-bridging mode. In this mode, the Y atoms are linked into a two-dimensional polymeric sheet parallel to the (010) plane. These sheets are in turn bridged via oxalate ligands. Both lengths and angles within the succinate anions exhibit normal values [Seguatni et al., 2004]. The oxalate ions locate on a center of inversion and act as double bidentate (tetradentate) ligands in a linear chain which connect two Y atoms in two different layers to form a 3D framework (Fig.2). The aqua ligands donate hydrogen atoms to carboxylate oxygen atoms O2 and O5 to form hydrogen bonds, which make a significant contribution to the stabilization of the crystal structure of the title yttrium compound.

### Experimental

A mixture of YCl_3_.6H_2_O (1.00 mmol, 0.30 g), oxalic acid (0.50 mmol, 0.05 g), succinic acid (0.50 mmol, 0.06 g), NaOH (2.00 mmol, 0.08 g) and H_2_O (10.0 ml) was heated in a 23 ml stainless steel reactor with a Teflon liner at 443 K for 48 h. The colorless plate-like crystals were filtered and washed with water and acetone. Yield: 26% based on Y.

### Refinement

H atoms attached to C atoms were included at calculated positions and treated as riding atoms [C—H = 0.97 Å and *U*_iso_(H) = 1.2*U*_eq_(C)]. The water H atoms were found in a diffrence map, relocated in idealized positions (O—H = 0.85 Å) and refined as riding atoms with *U*_iso_(H) = 1.5*U*_eq_(O).

### Crystal data

**Table:**

[Y_2_(C_4_H_4_O_4_)_2_(C_2_O_4_)(H_2_O)_2_]	*Z* = 1
*M**_r_* = 534.02	*F*(000) = 262
Triclinic, *P*1	*D*_x_ = 2.345 Mg m^−^^3^
Hall symbol: -p 1	Mo *K*α radiation, λ = 0.71073 Å
*a* = 6.610 (2) Å	Cell parameters from 336 reflections
*b* = 7.689 (3) Å	θ = 2.1–27.8°
*c* = 8.018 (3) Å	µ = 7.71 mm^−^^1^
α = 101.589 (5)°	*T* = 295 K
β = 101.843 (4)°	Plate, colorless
γ = 101.492 (5)°	0.21 × 0.18 × 0.09 mm
*V* = 378.2 (2) Å^3^	

### Data collection

**Table:**

Bruker APEXII CCD area-detector diffractometer	1482 independent reflections
Radiation source: fine-focus sealed tube	1376 reflections with *I* > 2σ(*I*)
graphite	*R*_int_ = 0.016
φ and ω scans	θ_max_ = 26.2°, θ_min_ = 2.7°
Absorption correction: multi-scan (*SADABS*; Sheldrick, 2003)	*h* = −8→7
*T*_min_ = 0.215, *T*_max_ = 0.505	*k* = −6→9
2108 measured reflections	*l* = −9→9

### Refinement

**Table:**

Refinement on *F*^2^	Secondary atom site location: difference Fourier map
Least-squares matrix: full	Hydrogen site location: inferred from neighbouring sites
*R*[*F*^2^ > 2σ(*F*^2^)] = 0.022	H-atom parameters constrained
*wR*(*F*^2^) = 0.059	*w* = 1/[σ^2^(*F*_o_^2^) + (0.0373*P*)^2^] where *P* = (*F*_o_^2^ + 2*F*_c_^2^)/3
*S* = 1.08	(Δ/σ)_max_ = 0.001
1482 reflections	Δρ_max_ = 0.60 e Å^−^^3^
119 parameters	Δρ_min_ = −0.54 e Å^−^^3^
0 restraints	Extinction correction: *SHELXL97* (Sheldrick, 2008), Fc^*^=kFc[1+0.001xFc^2^λ^3^/sin(2θ)]^-1/4^
Primary atom site location: structure-invariant direct methods	Extinction coefficient: 0.017 (3)

### Special details

**Table:**

Geometry. All e.s.d.'s (except the e.s.d. in the dihedral angle between two l.s. planes) are estimated using the full covariance matrix. The cell e.s.d.'s are taken into account individually in the estimation of e.s.d.'s in distances, angles and torsion angles; correlations between e.s.d.'s in cell parameters are only used when they are defined by crystal symmetry. An approximate (isotropic) treatment of cell e.s.d.'s is used for estimating e.s.d.'s involving l.s. planes.
Refinement. Refinement of *F*^2^ against ALL reflections. The weighted *R*-factor *wR* and goodness of fit *S* are based on *F*^2^, conventional *R*-factors *R* are based on *F*, with *F* set to zero for negative *F*^2^. The threshold expression of *F*^2^ > σ(*F*^2^) is used only for calculating *R*-factors(gt) *etc*. and is not relevant to the choice of reflections for refinement. *R*-factors based on *F*^2^ are statistically about twice as large as those based on *F*, and *R*- factors based on ALL data will be even larger.

### Fractional atomic coordinates and isotropic or equivalent isotropic displacement parameters (Å^2^)

**Table:**

	*x*	*y*	*z*	*U*_iso_*/*U*_eq_	
Y	0.53610 (4)	0.62226 (3)	0.80635 (3)	0.01351 (12)	
C1	0.1559 (4)	0.3484 (3)	0.7710 (3)	0.0152 (5)	
C2	−0.0273 (4)	0.1915 (4)	0.7540 (4)	0.0252 (6)	
H2A	−0.0878	0.2200	0.8536	0.030*	
H2B	0.0271	0.0844	0.7616	0.030*	
C3	−0.2057 (4)	0.1422 (4)	0.5858 (4)	0.0216 (6)	
H3B	−0.1424	0.1425	0.4868	0.026*	
H3A	−0.2900	0.0186	0.5697	0.026*	
C4	−0.3519 (4)	0.2691 (4)	0.5834 (3)	0.0181 (6)	
C5	0.3783 (4)	0.9630 (4)	0.9836 (3)	0.0161 (5)	
O1	0.2979 (3)	0.4094 (2)	0.9192 (2)	0.0192 (4)	
O2	0.1803 (3)	0.4203 (3)	0.6480 (2)	0.0213 (4)	
O3	−0.3798 (4)	0.3458 (3)	0.7273 (3)	0.0332 (5)	
O4	−0.4457 (3)	0.2882 (3)	0.4388 (3)	0.0330 (5)	
O5	0.3011 (3)	0.7981 (2)	0.8991 (3)	0.0214 (4)	
O6	0.2800 (3)	1.0725 (2)	1.0435 (3)	0.0210 (4)	
O7	0.8645 (3)	0.6937 (3)	0.7236 (3)	0.0280 (5)	
H7A	0.8427	0.6564	0.6127	0.042*	
H7B	0.9949	0.7307	0.7811	0.042*	

### Atomic displacement parameters (Å^2^)

**Table:**

	*U*^11^	*U*^22^	*U*^33^	*U*^12^	*U*^13^	*U*^23^
Y	0.01401 (16)	0.01412 (16)	0.01224 (16)	0.00374 (10)	0.00208 (9)	0.00406 (10)
C1	0.0149 (12)	0.0151 (13)	0.0164 (12)	0.0070 (10)	0.0039 (10)	0.0029 (10)
C2	0.0170 (13)	0.0255 (16)	0.0329 (15)	0.0021 (11)	0.0000 (12)	0.0171 (12)
C3	0.0149 (13)	0.0161 (14)	0.0275 (15)	0.0002 (10)	0.0005 (11)	−0.0001 (11)
C4	0.0177 (13)	0.0132 (13)	0.0199 (13)	−0.0011 (10)	0.0002 (10)	0.0059 (10)
C5	0.0145 (13)	0.0172 (13)	0.0169 (12)	0.0033 (10)	0.0024 (10)	0.0071 (10)
O1	0.0187 (9)	0.0201 (10)	0.0139 (9)	−0.0003 (8)	−0.0016 (7)	0.0044 (7)
O2	0.0216 (10)	0.0232 (10)	0.0162 (9)	−0.0001 (8)	0.0013 (7)	0.0076 (8)
O3	0.0434 (13)	0.0273 (12)	0.0304 (11)	0.0170 (10)	0.0105 (10)	0.0013 (9)
O4	0.0275 (11)	0.0441 (14)	0.0311 (11)	0.0075 (10)	0.0012 (9)	0.0253 (10)
O5	0.0160 (9)	0.0153 (10)	0.0290 (10)	0.0017 (7)	0.0049 (8)	−0.0004 (8)
O6	0.0164 (9)	0.0165 (10)	0.0301 (10)	0.0051 (8)	0.0074 (8)	0.0036 (8)
O7	0.0167 (9)	0.0412 (13)	0.0207 (10)	0.0033 (9)	0.0032 (8)	0.0012 (9)

### Geometric parameters (Å, °)

**Table:**

Y—O1	2.4755 (18)	C2—H2A	0.9700
Y—O1^i^	2.3319 (19)	C2—H2B	0.9700
Y—O2	2.4658 (19)	C3—C4	1.504 (4)
Y—O3^ii^	2.303 (2)	C3—H3B	0.9700
Y—O4^iii^	2.218 (2)	C3—H3A	0.9700
Y—O5	2.3876 (19)	C4—O4	1.249 (3)
Y—O6^iv^	2.3583 (19)	C4—O3	1.253 (3)
Y—O7	2.391 (2)	C5—O6	1.243 (3)
Y—Y^i^	4.0005 (11)	C5—O5	1.258 (3)
C1—O2	1.246 (3)	C5—C5^iv^	1.544 (5)
C1—O1	1.287 (3)	O7—H7A	0.8495
C1—C2	1.491 (4)	O7—H7B	0.8503
C2—C3	1.522 (4)		
			
O4^iii^—Y—O3^ii^	106.90 (8)	O5—Y—Y^i^	86.67 (5)
O4^iii^—Y—O1^i^	165.91 (7)	O7—Y—Y^i^	123.69 (5)
O3^ii^—Y—O1^i^	78.99 (7)	O2—Y—Y^i^	84.57 (5)
O4^iii^—Y—O6^iv^	89.63 (8)	O1—Y—Y^i^	32.56 (4)
O3^ii^—Y—O6^iv^	137.24 (8)	C1—Y—Y^i^	58.88 (5)
O1^i^—Y—O6^iv^	77.83 (7)	O2—C1—O1	118.6 (2)
O4^iii^—Y—O5	82.76 (8)	O2—C1—C2	123.3 (2)
O3^ii^—Y—O5	151.09 (8)	O1—C1—C2	118.1 (2)
O1^i^—Y—O5	98.21 (7)	O2—C1—Y	59.11 (13)
O6^iv^—Y—O5	68.11 (6)	O1—C1—Y	59.69 (12)
O4^iii^—Y—O7	76.30 (8)	C2—C1—Y	173.79 (19)
O3^ii^—Y—O7	74.60 (8)	C1—C2—C3	115.8 (2)
O1^i^—Y—O7	93.43 (7)	C1—C2—H2A	108.3
O6^iv^—Y—O7	71.50 (7)	C3—C2—H2A	108.3
O5—Y—O7	134.23 (7)	C1—C2—H2B	108.3
O4^iii^—Y—O2	74.88 (7)	C3—C2—H2B	108.3
O3^ii^—Y—O2	79.10 (7)	H2A—C2—H2B	107.4
O1^i^—Y—O2	119.12 (6)	C4—C3—C2	114.3 (2)
O6^iv^—Y—O2	143.65 (7)	C4—C3—H3B	108.7
O5—Y—O2	77.31 (7)	C2—C3—H3B	108.7
O7—Y—O2	132.82 (7)	C4—C3—H3A	108.7
O4^iii^—Y—O1	126.14 (7)	C2—C3—H3A	108.7
O3^ii^—Y—O1	75.63 (8)	H3B—C3—H3A	107.6
O1^i^—Y—O1	67.40 (7)	O4—C4—O3	123.0 (3)
O6^iv^—Y—O1	125.61 (6)	O4—C4—C3	118.9 (3)
O5—Y—O1	76.79 (7)	O3—C4—C3	118.0 (2)
O7—Y—O1	147.15 (7)	O6—C5—O5	127.1 (2)
O2—Y—O1	52.31 (6)	O6—C5—C5^iv^	116.6 (3)
O4^iii^—Y—C1	100.33 (8)	O5—C5—C5^iv^	116.3 (3)
O3^ii^—Y—C1	74.65 (8)	C1—O1—Y^i^	151.33 (16)
O1^i^—Y—C1	93.56 (7)	C1—O1—Y	93.65 (15)
O6^iv^—Y—C1	141.99 (7)	Y^i^—O1—Y	112.60 (7)
O5—Y—C1	76.83 (7)	C1—O2—Y	95.19 (15)
O7—Y—C1	146.47 (7)	C4—O3—Y^v^	129.0 (2)
O2—Y—C1	25.70 (7)	C4—O4—Y^iii^	165.24 (19)
O1—Y—C1	26.66 (7)	C5—O5—Y	118.76 (16)
O4^iii^—Y—Y^i^	158.49 (6)	C5—O6—Y^iv^	120.22 (16)
O3^ii^—Y—Y^i^	74.64 (6)	Y—O7—H7A	110.0
O1^i^—Y—Y^i^	34.84 (4)	Y—O7—H7B	133.6
O6^iv^—Y—Y^i^	103.73 (5)	H7A—O7—H7B	115.4

Symmetry codes: (i) −*x*+1, −*y*+1, −*z*+2; (ii) *x*+1, *y*, *z*; (iii) −*x*, −*y*+1, −*z*+1; (iv) −*x*+1, −*y*+2, −*z*+2; (v) *x*−1, *y*, *z*.

### Hydrogen-bond geometry (Å, °)

**Table:**

*D*—H···*A*	*D*—H	H···*A*	*D*···*A*	*D*—H···*A*
O7—H7A···O2^vi^	0.85	2.02	2.867 (5)	175
O7—H7B···O5^ii^	0.85	1.96	2.812 (4)	175

Symmetry codes: (vi) −*x*+1, −*y*+1, −*z*+1; (ii) *x*+1, *y*, *z*.
